# The New Medical College

**Published:** 1879-01-20

**Authors:** 


### EDITORIAL AND MISCELLANEOUS.

### *THE NEW MEDICAL COLLEGE.*

For many years suggestions have reached us from all portions of the South, relative to the establishment, in the city of Atlanta, of a first class Medical College.

The establishment of another Medical Institution in the face of the assertion, so often made, that too many schools already exist, has not been determined upon without careful and mature deliberation, and has not been prompted by any spirit of opposition to other schools. True emulation and honorable competition in the effort to increase the facilities and elevate the standard of medical education in the Southern section of the Union, are deemed worthy and laudable motives,

The central position of Atlanta; its accessibility; its favorable location as a financial and commercial centre, together with its exemption from devastating epidemics to mar its prosperity, or disturb its enterprises, all combine in a peculiar degree to fit our citty for the high position, to which she is destined to attain, of a great medical centre of the South.

Taking this view of Atlanta's future, the founders have, in connection with some of the most distinguished citizens of Georgia, formed a Board of Trustees and procured a Charter. The Trustees will, at an early day, organize and proceed to elect suitable gentlemen as a Faculty for the Institution, which will be known and recognized as the SOUTHEN MEDICAL COLLEGE.

It is the desire of the Trustees to embrace in the curriculum of studies to be taught in the Institution those of the best organized Medical Schools of the country, and have, therefore, determined upon the following Chairs:

*Professorships.*—1. The Theory and Practice of Medicine; 2. General and Pathological Anatomy; 3. The Principles and Practice of Surgery; 4. Ophthalmic Surgery and Diseases of the Ear and Throat; 5. Physiology and Hygiene; 6. Materia Medica, Medical Pharmacy and Therapeutics; 7. Dermatology and Medical Literature; 8. Chemistry and Toxicology; 9. The Principles and Practice of Obstetrics, Clinical Gynæcology and Medical Ethics; 10. Diseases of Women and Children and Operative Gynæcology; Demonstrator of Anatomy; Assistant Demonstrator of Anatomy.

In addition to the above, which will coustitute the Faculty proper. Clinical Lectures will be given by the Professors, and competent medical gentlemen will be appointed to lecture upon important collateral branches, as follows:

*Lecturers.*—A Lecturer on Medical Botany and Indigenous Remedies of the United States; A Lecturer on Genito-Urinary and Venerial Diseases; A Lecturer on Minor Surgery and Assistant to Professor of Surgery; A Lecturer on Electro-Therapeutics.

It will be seen that it is the design to cover the entire ground occupied by the most advanced Medical Colleges of this country.

The Trustees, we learn, will, between the 1st and 10th of March next, proceed to elect Teachers to the Chairs above mentioned, that they may have ample time to prepare for the opening of the College next Fall.

We are requested to say that, for the present, applicants for Chairs, and parties desiring information, may address the Managing Editor of this Journal, who consents to act as Corresponding Secretary of the Board for the time being.

### *OUR JOURNAL.*

At the risk of being considered fickle, we have resumed the single page form in our journal. It was found best to do so on account of the extra and unnecessary trouble which the double column gave the printer.

The type in the present journal is better and is one size smaller, which with the eight pages added, more than compensates for difference in size of page.

In all respects we think the present journal is a decided improvement. Our list should now rapidly increase. See our premium offer.

